# The Adventures of Amaru: Integrating Learning Tasks Into a Digital Game for Teaching Children in Early Phases of Literacy

**DOI:** 10.3389/fpsyg.2018.02531

**Published:** 2018-12-14

**Authors:** Gilberto Nerino de Souza, Yvan Pereira dos Santos Brito, Myenne Mieko Ayres Tsutsumi, Leonardo Brandão Marques, Paulo Roney Kilpp Goulart, Dionne Cavalcante Monteiro, Ádamo Lima de Santana

**Affiliations:** ^1^Technology Institute, Postgraduate Program in Electrical Engineering, Federal University of Pará, Belém, Brazil; ^2^Exact and Natural Sciences Institute, Computer College, Federal University of Pará, Belém, Brazil; ^3^Graduate Program in Neurosciences and Behavior, Department of Behavior Theory and Research, Federal University of Pará, Belém, Brazil; ^4^Center of Excellence for Social Technologies (NEES), Education Center, Federal University of Alagoas, Maceió, Brazil

**Keywords:** digital game, teaching, reading, matching-to-sample, narrative and gameplay

## Abstract

In low-income countries, the history of academic failure is a liability for children acquiring literacy skills. It is thus important to develop strategies that motivate and focus these students on specific strategies to learn to read. Digital games can be useful in motivating students and assisting teachers in the teaching-learning process, but there are few interactive tools that effectively integrate tasks of direct instruction and good gameplay. This technical report describes an interactive digital game to engage students in the initial phase of reading skills acquisition, whose design incorporates evidence-based procedures. The game, called “The Adventures of Amaru,” aims to promote word coding-decoding skills and vocabulary growth through teaching trials. We discuss the adaptation of reading teaching curricula, their limitations and future implications of the use of this game by children from a low-income background.

## Introduction

Adopting new technologies of gaming as part of the teaching-learning process can promote engagement in academic activities with attractive experiences that meet educational needs and lead to better outcomes for the students (Katsikitis et al., 2014; Abdul Jabbar and Felicia, 2015; Ke, 2016). This can be particularly significant for children of developing countries such as Brazil, where the average performance in reading of students is meaningfully below the Organization for Economic Cooperation and Development (OECD) average: with 407 points, compared to the average of 493 points on the PISA (Program for International Student Assessment) standardized test. An aggravating factor is that the share of low performers in reading in Brazil has remained the same since 2009 (OECD, 2015). Besides that, according to the Brazilian Institute of Geography and Statistics (IBGE), in 2011 more than 4 million children aged between 5 and 7 years were illiterate, and many of the students in the subsequent grades did not have basic reading skills (IBGE, 2011). The experience of academic failure has negative impacts on reading performance and can cause demotivation, school dropout, and reading non-comprehension (Pajares, 2003; Neild et al., 2008; López et al., 2017).

One workaround indicated by researchers is the positive influence of direct instruction procedures to foster the learning of basic reading skills, such as the teaching program called Learning to Read and Write in Small Steps (ALEPP – acronym in Portuguese) (De Souza et al., 2009; Rose et al., 2012). The ALEPP curricula are aimed at identifying and remediating the basic reading skills of students with a history of academic failure in regular classes. This teaching program uses the consolidated discrete-trial procedure Match-to-Sample (MTS) to promote learning of the initial basic reading skills and vocabulary formation (Perfetti and Hogaboam, 1975). When integrated into a computerized learning path manager, this program allows for the monitoring of progress through educational units (set of activities to be taught) which, in turn, allows for analysis of the learning needs of each student (Orlando, 2009). However, ALEPP presents the teaching tasks (the same used in basic cognitive and behavioral process research) in a classic MTS layout and structure because of the growing and consistent learning curve, which often leads students to boredom and considerable abandon rates, as discussed in a series of doctoral and masters theses that tested this procedure in Brazil.^1^

In this context, researchers (Battaiola et al., 2008; McGonigal, 2011; Kim et al., 2015; Cezarotto and Battaiola, 2016) also assert that the narrative of a game and its characteristics can be determinant in maintaining the player’s engagement. Generally, learning is sustained by motivational applications and by conventional methods (Marques et al., 2013; Mourgues et al., 2016), but the students prefer educational methods based on games, rather than other applications that do not have game characteristics (Sarmanho, 2012; Hwang and Wang, 2016).

Some studies (Smith et al., 2013; Ronimus et al., 2014; Hwang and Wang, 2016) have had positive results in improving teaching using motivational aspects. Smith et al. (2013) investigated the learning and engagement of Chinese university students in teaching English vocabulary; there were indications of learning in computer games with better knowledge gains than traditional methods. Hwang and Wang (2016) developed an educational game with the goal of observing learning and engagement in language teaching; the study confirmed that games could engage students in the search for better answers. In the research developed by Ronimus et al. (2014), the objective was to help children with dyslexia identify the sounds of letters and words; the effects of reward, challenges, and engagement of children in educational games have showed that digital games could be good tools for aiding literacy.

In the domain of stimuli association using the MTS procedure for improving reading, the game used by Marques et al. (2013) and Siqueira et al. (2012) has a story wherein the player walks through the stages in a style similar to the classic RPGs (Role-Playing Games), such as Zelda^TM^, Chrono Trigger^TM^ and Phantasy Star^TM^. Marques et al. (2013) used the digital game with a playful plot and a progressive transition of learning tasks based on the student’s performance. The learning trials were presented as a solution to puzzles and the discovery of paths. The authors indicated that the use of interactive multimedia such as games and web applications stimulated the student’s interests. In this protocol, the player must accept some challenges in the course of the game to complete a set of items. However, at the time of learning words, the player is presented in the style of classic MTS trials with little interactivity. This can lead the player to “escape” these challenges by looking for other challenges inherent in the game itself. There are tools that have several forms of interactivity and gameplay, but the mini-games do not interconnect in a narrative or story that could aid in the engagement of the player for a longer time in the teaching, as does the game used by researchers Richardson and Ronimus (Richardson and Lyytinen, 2014; Ronimus and Richardson, 2014).

The objective of this technology report is to present a digital game called “The Adventures of Amaru” (in Portuguese – “As Aventuras de Amaru”), that addresses these issues by means of an innovative game-based design, and assists written word recognition using image-text-speech relations. The MTS procedure was embedded in a personalized learning path and game-like activities in the hope of increasing engagement and learning. The game uses features that can be attractive to children, such as story and interesting characters, rewards after the completion of challenges and fewer repetitive mini-games (Ronimus et al., 2014). Additionally, the integration and inclusion of trials and stimuli are intended to be relatively simple for a teacher trained in the behavioral analysis process.

## Materials and Methods

**FIGURE 1:**
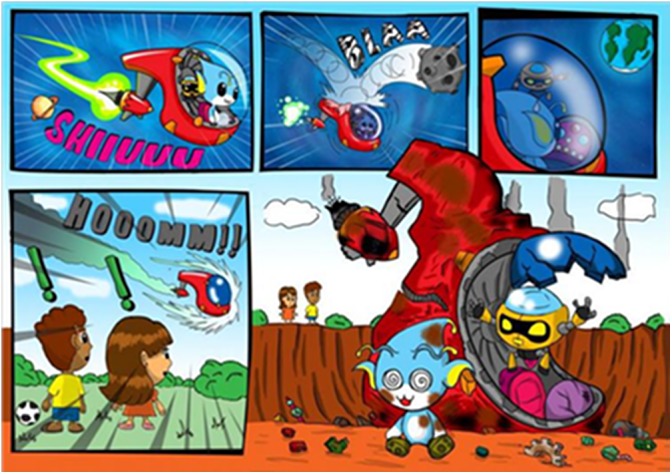
After a meteor shower, Amaru was forced to land on earth, where he meets two kids that soon befriend him. Amaru has a faithful squire who accompanies him, the robot Urama.

The digital game used in the research was built using the Unity 3D engine (Unity, 2018) and C# programming language, with the mechanics of platform games (e.g., Super Mario Bros^TM^, Sonic^TM^, and Alex Kid^TM^). The game presents MTS teaching trials integrated to a story that revolves around the character named Amaru, along with his robot friend named Urama. The 3D objects were modeled in the Blender tool (Blender, 2018), with main characters and the 2D objects edited in the GIMP (GNU Image Manipulation Program) tool (Peck, 2006; GIMP, 2013). The graphic arts of the characters were inspired by virtual pets generally found in cartoons (e.g., Pokemon^TM^, Digimon^TM^, and Medabot^TM^), to further appeal to children. Moreover, the sound, audio, and image aspects of this game were built by a team specialized in multimedia. The game narrative was created with gamification aspects, as shown in the story clipping in Figure 1.

In the context of the game narrative, the teaching trials are intended to teach the character to communicate in the local language with the two children presented at the beginning of the story, hence Amaru’s need to learn to read the objects in the new planet to fix his spaceship, in this case, words written in Brazilian Portuguese.

A test conducted with an early prototype of the game proved to be valuable, allowing us to improve its utility by better understanding the features of the game, fixing bugs and making software improvements, such as the insertion of new mini-games and types of tasks (de Souza et al., 2017).

The basic skills that the ALEPP promotes are words and syllables written to spoken bidirectional relations through MTS trials, similar to decoding skill as (context-free) word recognition or phonics. The MTS is structured as a discrete trial task with one stimulus, functioning as a model and two or more comparisons stimuli, from which the subject can choose, promoting a conditionally discriminative behavior (Sidman and Tailby, 1982). Then, the subject can identify which is the correct option to choose (i.e., the written word “*cake*”) in the presence of a certain model (i.e., the spoken word “*cake*”).

Additionally, the task ordering and conditionalities (alternative options that are displayed) ensure the evaluation of a basic level of comprehension by two MTS tasks: (1) figure-word recognition test, when the spoken word is the model and different figures are the comparisons; (2) emergent behavior, based on the assessment of non-direct relations between written words and figures that illustrate it. The emergent behavior means that to demonstrate mastery of this skill, the student needs first to relate the spoken word with the figure, and, afterward, relate the written word with its spoken counterpart. A form of conceptual learning occurs if the relation between the written word and the figure emerges, according to the equivalence paradigm (Sidman et al., 1989; Sidman, 2000).

The order of tasks implemented in the game promotes equivalence relations among words via procedures of modalities association, such as written, spoken, figures (representative) and syllables (as word components). These word modality associations are always based on MTS and CRMTS (Constructed Response – MTS); the latter for the training of composition of words by the correct composition of letters and syllables. The behavioral teaching procedures programmed in the ALEPP curriculum focus on word decoding and in reinforcing the Brazilian Portuguese vocabulary. Table 1 presents examples of tasks that promote the word modality relationships used in the game.

**Table 1:** Types of equivalence relations of the MTS and CR-MTS procedure used in this proposal.

Trial illustration	Trial description	Trial illustration	Trial description
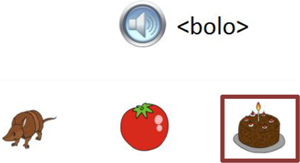	Identification of the type of relation (nomenclature): AB. Based on a sound instruction, the student must choose the picture of a “bolo” (cake).	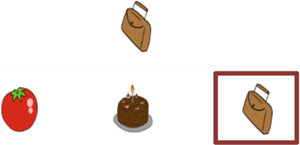	Identification of the type of relation (nomenclature): BB. Based on the picture of a “mala” (suitcase), the student must choose the picture of a “mala” (suitcase).
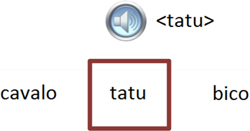	Identification of the type of relation (nomenclature): AC. Based on a sound instruction, the student must choose the word “tatu” (armadillo)	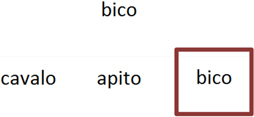	Identification of the type of relation (nomenclature): CC. Based on the word “bico” (beak), the student must choose the word “bico” (beak).
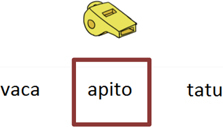	Identification of the type of relation (nomenclature): BC. Based on the picture of an “apito” (whistle), the student must choose the word “apito” (whistle).	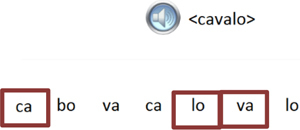	Identification of the type of relation (nomenclature): AE. Based on a sound instruction of word “cavalo” (horse) the student must choose the syllables in the correct order: ca - va - lo.
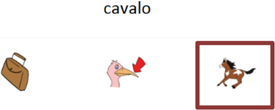	Identification of the type of relation (nomenclature): CB. Based on a written instruction, he student must choose the picture of a “cavalo” (horse).	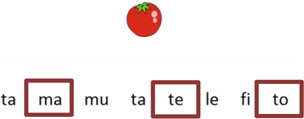	Identification of the type of relation (nomenclature): BE. Based on the picture of a “tomate” (tomato), the student must choose the syllables in the correct order: to - ma – te.
		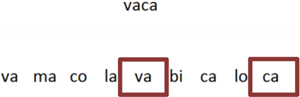	Identification of the type of relation (nomenclature): CE. Based on the word “vaca” (cow), the student must choose the syllables in the correct order: va - ca.


## The Proposed Teaching Digital Game

The digital teaching game produced in this work is named “The Adventures of Amaru” and can be used for both training and teaching words, as well as for evaluation (similar to a test). For evaluation purposes, however, there is no correct or error feedback, and the player only has one chance to succeed in each trial.

**FIGURE 2:**
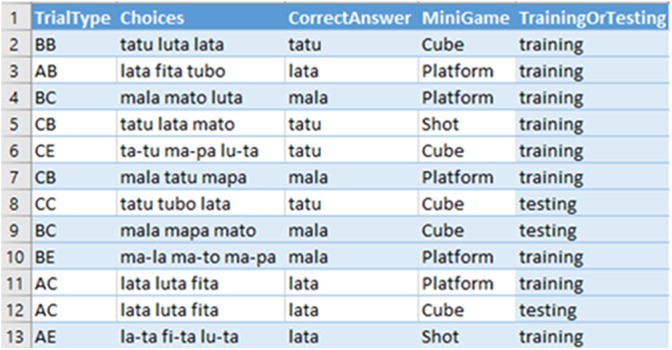
CSV file configuration of a set of trials inserted for in-game presentation.

To aid in the insertion of trials by the teacher, an auxiliary tool was developed that allows the inclusion of a CSV (Comma-Separated Values) file, where each row is a trial and each column is an attribute of this trial, as shown in Figure 2. The first column indicates the task type, or the word modality relation being taught. The game makes a uniform division of trials per stage: for example, with a set of 45 trials inserted in the game, each stage will have 9 trials, following the order stipulated in the CSV file. The images, sounds, and texts are stored in folders with their respective settings, which allows the easy inclusion and removal of the assets used to compose a trial.

**FIGURE 3:**
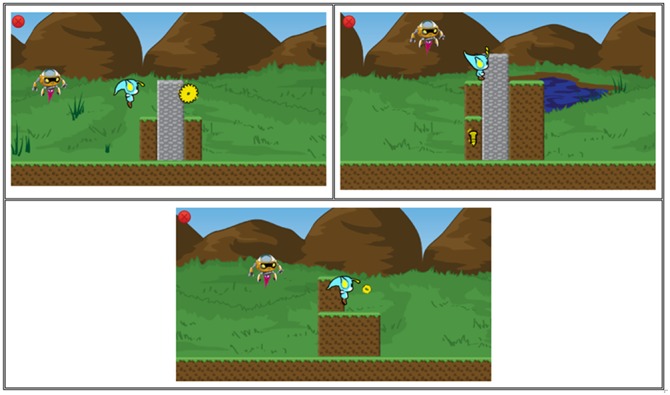
Game items and obstacles between trials.

**FIGURE 4:**
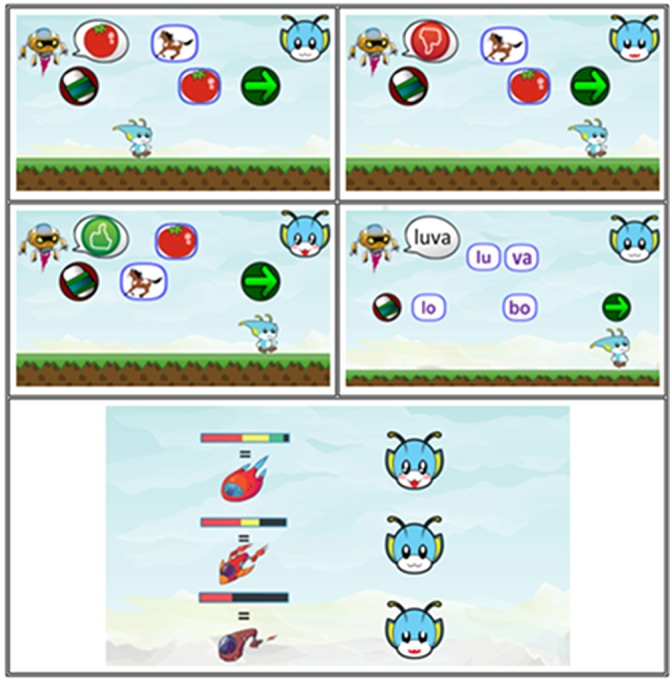
Game tutorial screens.

The main goal of the player, playing as Amaru, is to rebuild the spaceship, and to do so he/she needs to collect items such as gears, bolts, and nuts in game stages. Each item has a specific score and, based on the items collected at the end of the journey, the player gains a ship, as shown in Figure 3. The rewards after performing an activity and collecting items are also gamification aspects of the MTS process. In the game, these items are found and the character is successful on the trial. For each wrong answer on a trial, the item to be collected undergoes depreciation, that is, if the player were to make only correct choices, the item will have a higher value, making a better-looking ship available at the end of the game. In training mode, the player has three chances to answer correctly on each trial. If the player chooses incorrectly three times on a given trial, the player advances to the next attempt, but does not gain any item to improve his ship. A tutorial at the beginning of the gameplay shows the commands, the player’s mission and the ships he/she can win based on the items collected, as shown in Figure 4.

**FIGURE 5:**
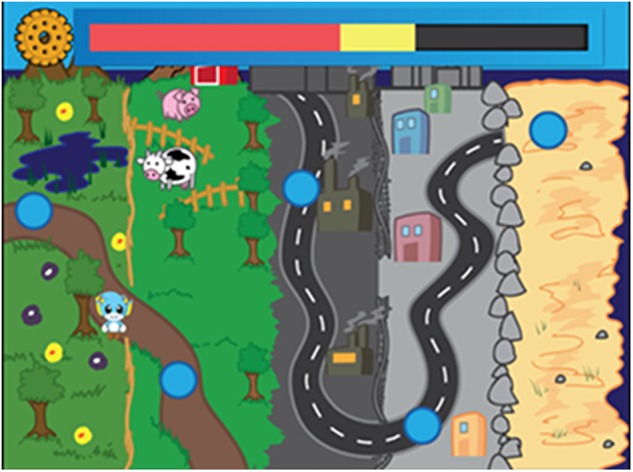
Mini-map between stages of the game, similar to a previous prototype (de Souza et al., 2017) (Reproduced with the consent of the IEEE copyright).

A stage in the game is basically composed of modified MTS trials that the player executes controlling an avatar (i.e., Amaru), with keyboard, mouse, joystick or computer touch-screen. The novel fun game features may only have a short-term positive impact (Ronimus et al., 2014). Soon after each trial, an obstacle arises that has the collectible item as a reward. This cycle repeats itself until the trials of that stage are finished. The game has five stages, set in the following environments: forest, countryside, city, industry, and beach. At the end of each stage, there is a screen transition that shows a mini-map and the progress of the score (Figure 5). This status display is a gamification feature that generates a sense of advancement and ultimate goal to be fulfilled.

**FIGURE 6:**
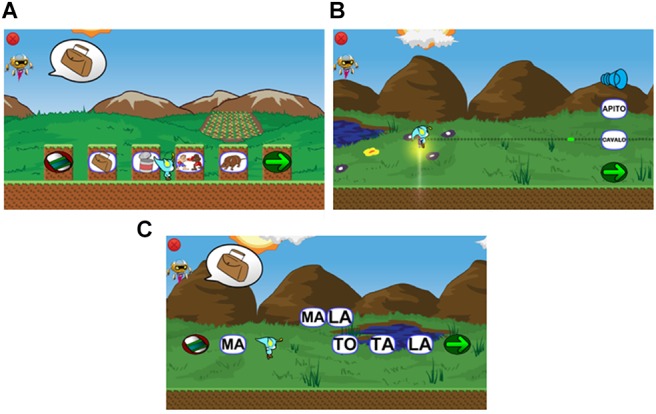
Trials presented in the game. **(A)** platform mini-game, **(B)** shooting mini-game and **(C)** cubes mini-game.

One of the characteristics of classic MTS trials in computing environments is that the person must select (i.e., clicking the mouse button) the option which he/she considers correct. In this game, different means are implemented for selecting the options (Figure 6). In the first kind of player-task interaction, the avatar must jump and touch one of the floating options above the screen. In the second kind, the player must jump on a platform to choose the option. In the third, the avatar can shoot with a projectile to hit the target options. The position of the options is shuffled trial-by-trial to avoid repeating positions of the correct choice and to stimulate the screening behavior of the students. These strategies can make the game more dynamic and less repetitive. Despite the response type variations on the stages, at the behavior control level, all the game mechanics used to promote the same learning relations are found in regular MTS.

**FIGURE 7:**
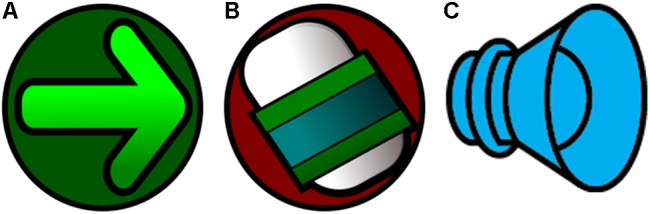
**(A)** image of the “arrow” that must be activated to confirm the option selected in the trial, **(B)** image of “eraser” to correct the option and redo the trial, **(C)** image of a “speaker” to hear the trial model/command again.

The character Urama is a helper avatar that has the role of presenting the reference model (the word in the format of speech, text or figure). The game provides positive feedback when the option is correct and a ‘try again’ feedback when it is incorrect. In order for Amaru to complete the task and move on to the next trial, the player must select the green arrow, Figure 7A, that indicates continuation of the gameplay, or he/she can redo the task by selecting the icon of an eraser, Figure 7B. In the case of trials that have sound (speech model), the player can select the option to listen again to the sound of the word dictated, Figure 7C.

**FIGURE 8:**
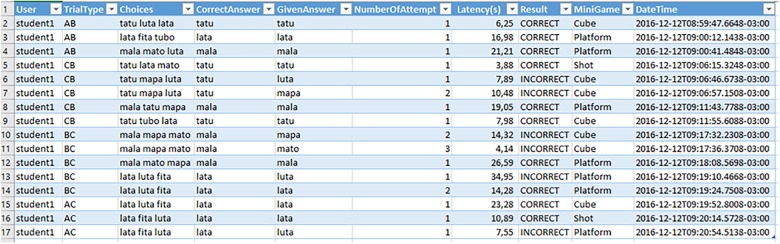
Log file generated at the end of the game.

At the end of the stages, the game generates a log file in tabulated format. The log arranges the trials configuration and the user responses (see in Figure 8), allowing further analysis by the teacher regarding the student’s progress.

**FIGURE 9:**
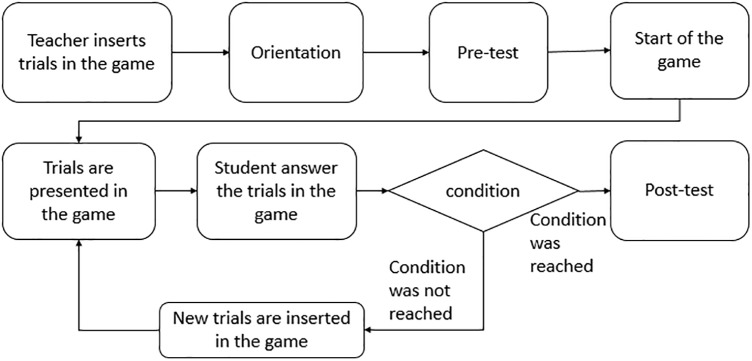
Flowchart with an example of teaching activity using the game.

Figure 9 illustrates the use of a teaching activity in an environment with teachers and students, using the game as an educational tool. At an early stage, the teacher inserts the trials in the game to be presented to the student. Students are then given guidance on how to use the software. In order to evaluate learning, a pre-test is applied. Trials are presented to the student individually, and if the student meets a condition, he/she may be presented with further trials that teach other words. For example, if the student achieves more than 70% of the correct trials, he/she advances to a new teaching stage. In the end, a post-test can be applied and compared to the pre-test; in this way, the teacher can identify improvements in the student’s learning.

## Discussion and Future Works

According to the Brazilian National Literacy Assessment, in 2016 more than 50% of children aged between five and eight had insufficient knowledge according to Bermúdez (2017). The northern region of Brazil was the most worrisome, with approximately 70% of school-age children presenting some difficulty in reading and writing (Bermúdez, 2017). “The Adventures of Amaru” is a game that uses evidence-based teaching procedures to help promote basic reading skills. This kind of technology can aid schools in low-income areas that do not have internet or updated computers. For example, schools in cities such as Belém, Brazil, have computers but do not have stable internet connections.

Using play tools to teach children to read increases the possibility of engaging them and even their learning. In the literature that investigates the use of games as a teaching tool, reviews show not only an increase in academic performance, but also improved motivation of the participants (Clark et al., 2016; Ke, 2016). The main application of a playful technology intended to teach reading is geared to the school environment, but not exclusively. Domestic use of the game can also be useful, as it exposes the child to a pleasurable activity that also teaches. The main advantage of educational games using artificial intelligence is the capacity of identifying student performance and, based on it, programming the ensuing tasks. This saves time for the educator, who can then serve as a mentor, and exposes the students to the training while maintaining motivation through engagement and introducing new challenges through novel tasks. Students who are in the initial stage of literacy can use this technology as a complementary tool for learning idioms. The teacher can use it both as a class activity and as homework. In a playful way, technology can teach, using the combination of images and sounds and through a paradigm that tries to understand human behavior and, as well, employ an approach that matches how effective teaching actually occurs.

The difference between “The Adventures of Amaru” and other computerized games designed for teaching lies in the approach of integrating the teaching procedure into a narrative and merging it with the gameplay. The items and stimuli are integrated with the story, making sense within the narrative, thus aiming to discourage the player from choosing to engage in the non-educational aspects of the game over the educational aspects. In contrast, other digital games (i.e., Tetris^TM^, Snake^TM^, and Pong^TM^) use gameplay disconnected from the story, and with extra classic mini-games as rewards to sustain the motivation.

“The Adventures of Amaru” presents the MTS trials as a series of continuing challenges, implemented as “mini-games” planned to entertain and teach at the same time. Additionally, the game’s architecture enables the creation and inclusion of new mini-games in future versions of the software, allowing new kinds of playful interactions. An additional advantage is that the game can be used at school or home, likely increasing its probability of use.

One strength of the game design is that it has the flexibility to be used with other languages and even different domain areas (e.g., math operations). Students with learning disabilities could also be benefited, since the game implements automated, instruction-free procedures, in a ludic context. For this reason, the game will be available and freely accessible to interested researchers accessing the web page, with the game installation step by step, the supplementary material, access to explanations videos and the current state of this research by the link http://linc.ufpa.br/amaru-mts/.

The project is heading toward the use of artificial intelligence techniques to produce new trials personalized for students. The authors intend to use assistive tools for the automatic generation of individualized reading tasks estimated by the performance of non-literate students.

Analysis of the learning of children in low-income areas can also be accomplished; it is possible that new data related to these experiments will be released at the end of this year. It is expected that, with further studies in this area, “The Adventures of Amaru” will be a valid, meaningful, and useful tool for the teaching-learning process.

This technology is a low-cost approach. Despite this, a major limitation is a need for financial resources to obtain a minimal structure, such as a computer, which not all children and schools are able to access, especially in low-income areas. In addition, the content that is presented in the game in its current version is preliminary and restricted to initial stages of literacy. There is a moderate amount of words or combinations of them. Further research can show and test the complexification the words to be taught.

## Author Contributions

GdS, MT, LM, PG, and DM made substantial contributions to the development of the concepts. GdS, MT, LM, PG, DM, and ÁS contributed to the drafting and approval of this manuscript and agreed to be held accountable for all aspects of this work. GdS and YB provided the computer programming of this work. GdS originated the project and coordinated the paper team.

## Conflict of Interest Statement

The authors declare that the research was conducted in the absence of any commercial or financial relationships that could be construed as a potential conflict of interest.

## Footnotes

1 The ALEPP curriculum has been developed by a group of researchers motivated to handle the performance literacy gap of brazilian children. These efforts details can be found on the SubProject 7A (Reading, writing, and mathematics in the classroom) from the “National Institute of Science and Technology on Behavior, Cognition, and Teaching/” Final Report (http://www.inctecce.com.br/images/annual-report/web_inct-ecce-7_high-2.pdf).
